# Normalization of Snai1-mediated vessel dysfunction increases drug response in cancer

**DOI:** 10.1038/s41388-024-03113-1

**Published:** 2024-08-02

**Authors:** Helene Hoffmann, Martin Wartenberg, Sandra Vorlova, Franziska Karl-Schöller, Matthias Kallius, Oliver Reinhardt, Asli Öztürk, Leah S. Schuhmair, Verena Burkhardt, Sabine Gätzner, Daniela Scheld, Rajender Nandigama, Alma Zernecke, Sabine Herterich, Süleyman Ergün, Andreas Rosenwald, Erik Henke

**Affiliations:** 1 Institute of Anatomy and Cell Biology, Universität Würzburg, Koellikerstrasse 6, 97070 Würzburg, Germany; 2 Graduate School of Life Science, Universität Würzburg, Josef-Schneider-Strasse 2, 97082 Würzburg, Germany; 3 Institute of Pathology, Universität Würzburg, and Comprehensive Cancer Center Mainfranken (CCCMF), Josef-Schneider-Strasse 2, 97082 Würzburg, Germany; 4 Institute of Tissue Medicine and Pathology (ITMP), Universität Bern, Murtenstrasse 31, 3008 Bern, Switzerland; 5 Institute of Experimental Biomedicine II, Universitätsklinikum Würzburg, Josef-Schneider-Strasse 2/D16, 97082 Würzburg, Germany; 6 Chair Tissue Engineering and Regenerative Medicine (TERM), Universitätsklinikum Würzburg, Roentgenring 11, 97070 Würzburg, Germany; 7 Zentrallabor, Universitätsklinikum Würzburg, Josef-Schneider-Strasse 2, 97082 Würzburg, Germany

**Keywords:** Cancer microenvironment, Tumour angiogenesis, Cancer therapeutic resistance, Metastasis

## Abstract

Blood vessels in tumors are often dysfunctional. This impairs the delivery of therapeutic agents to and distribution among the cancer cells. Subsequently, treatment efficacy is reduced, and dose escalation can increase adverse effects on non-malignant tissues. The dysfunctional vessel phenotypes are attributed to aberrant pro-angiogenic signaling, and anti-angiogenic agents can ameliorate traits of vessel dysfunctionality. However, they simultaneously reduce vessel density and thereby impede drug delivery and distribution. Exploring possibilities to improve vessel functionality without compromising vessel density in the tumor microenvironment, we evaluated transcription factors (TFs) involved in epithelial-mesenchymal transition (EMT) as potential targets. Based on similarities between EMT and angiogenic activation of endothelial cells, we hypothesized that these TFs, Snai1 in particular, might serve as key regulators of vessel dysfunctionality. In vitro, experiments demonstrated that Snai1 (similarly Slug and Twist1) regulates endothelial permeability, permissiveness for tumor cell transmigration, and tip/stalk cell formation. Endothelial-specific, heterozygous knock-down of Snai1 in mice improved vascular quality in implanted tumors. This resulted in better oxygenation and reduced metastasis. Notably, the tumors in Snai1KD mice responded significantly better to chemotherapeutics as drugs were transported into the tumors at strongly increased rates and more homogeneously distributed. Thus, we demonstrate that restoring vessel homeostasis without affecting vessel density is feasible in malignant tumors. Combining such vessel re-engineering with anti-cancer drugs allows for strategic treatment approaches that reduce treatment toxicity on non-malignant tissues.

## Introduction

Systemic anti-cancer drugs often accumulate at significantly lower doses in the malignant target tissue than in non-neoplastic tissues [1–3]. Increased adverse side effects and even premature termination of therapy as a result of intolerable treatment toxicity are negative clinical consequences. The dysfunctional vasculature in the tumor microenvironment (TME) has been identified as a major contributing factor for insufficient drug delivery to and distribution among cancer cells [4]. Consequently, improving the defective vasculature phenotype and function has been proposed as a promising way to enhance tumor drug delivery, thereby increasing therapeutic efficacy, reducing side effects, and preventing premature treatment termination [5, 6]. Sustained aberrant pro-angiogenic signaling in the microenvironment with unbalanced, abundant expression of neoangiogenesis-stimulating cytokines and growth factors (GFs) has been identified as a crucial factor for defective/dysfunctional vasculature in the TME of malignancies. However, such inflammatory mediators are not only causal for the dysfunctional state of tumor vasculature but also needed to form and maintain an adequately dense vasculature. Thus, targeting major pro-angiogenic signaling pathways not only has the favorable potential to improve vessel functionality but also the adverse effect of reducing vessel density. Consequently, anti-angiogenic therapies often result in tumor hypoxia and detrimental reduction of intratumoral drug supply, which can even lead to therapeutic failure [7, 8].

While quantitative and functional vascular inadequacy is common in malignant neoplasia, a remarkable heterogeneity exists with respect to microvessel density (MVD), vessel size, vascular integrity, and pericyte coverage, even in cancers originating from the same tissue [9–11]. This naturally occurring diversity is a suitable starting point for understanding the mechanisms that regulate vascular phenotypes and morphology. Tumor angiogenesis is stimulated by many different growth factors and chemokines, like VEGF-A, PlGF, bFGF, angiopoietins, and Il-8, and is strongly influenced by the inflammatory microenvironment of the respective tumor. The spectra and ratio of pro-angiogenic factors secreted by cancer, stromal, and immune cells vary strongly between tumors [10], resulting in a complex balance of stimulating and inhibitory influences on endothelial cells. The endothelial response to these pro- and anti-angiogenic signals is then translated into the expression of different transcription factors (TFs). Consequently, various TFs are upregulated in endothelial cells of malignant tumors compared to non-malignant tissues [12–14]. Individual TFs most likely control different processes during tumor angiogenesis—an assumption supported by diverse vascular defects observed during ontogenesis in TF-knockout animals. For some TFs (like Id1/Id3 [15], Erg [16], and Sox18 [17]), it has been demonstrated that defects in tumor angiogenesis parallel ontogenetic defects.

Epithelial-mesenchymal transition (EMT) is essential to many physiological processes like embryonic development and is also regarded as a key mechanism in cancer progression [18]. Carcinoma cells can shift their phenotype to a more mesenchymal phenotype, thereby increasing motility and invasiveness [19]. TFs like Snai1, Slug, and Twist1 have been demonstrated to be key regulators of EMT in carcinomas [20–22]. A similar process is observed in endothelial cells (endothelial-mesenchymal transition, EndMT) [23]. TFs similarly govern the EndMT program, and again Snai1, Slug, and Twist1 appear to play a central role [24–28]. Slug seems to initiate parts of this EndMT program as an endothelial activator in tumor angiogenesis [29]. Furthermore, endothelial-specific knock-out of Snai1 delays the appearance of experimental breast tumors and influences associated stroma formation, immune cell infiltration, and tumor phenotype [30]. However, so far, studies have focused on the role of endothelial TFs for tumor initiation and manifestation; an understanding of how these TFs affect vessel functionality and thereby hamper drug delivery to and distribution in neoplasia is yet to be unveiled.

In this study, we demonstrated that Snai1 is upregulated in the vascular endothelium of carcinomas. Such constant, aberrant signaling resulted in permanent upregulation of EndMT-related pathways and contributed to vascular dysfunctionality. In murine models, even partial reduction of endothelial Snai1-signaling improved tumor vessel architecture, maturity, and patency. These changes directly resulted in a reduction of malignant features and strongly improved accumulation of and response to chemotherapy. Importantly, these results stood in stark contrast to the effects of anti-angiogenic VEGF-ablation therapy, which, by reducing vascularization, increased features of malignancy and did not improve drug supply to the tumor. In conclusion, EMT-regulating TFs play a significant role in regulating dysfunctional blood vessel phenotypes in cancer, and targeting them might serve as a therapeutic means to achieve normalized blood vessel homeostasis.

## Results

### EMT-inducing TFs are upregulated in the endothelium of neoplasia

**Fig. 1:**
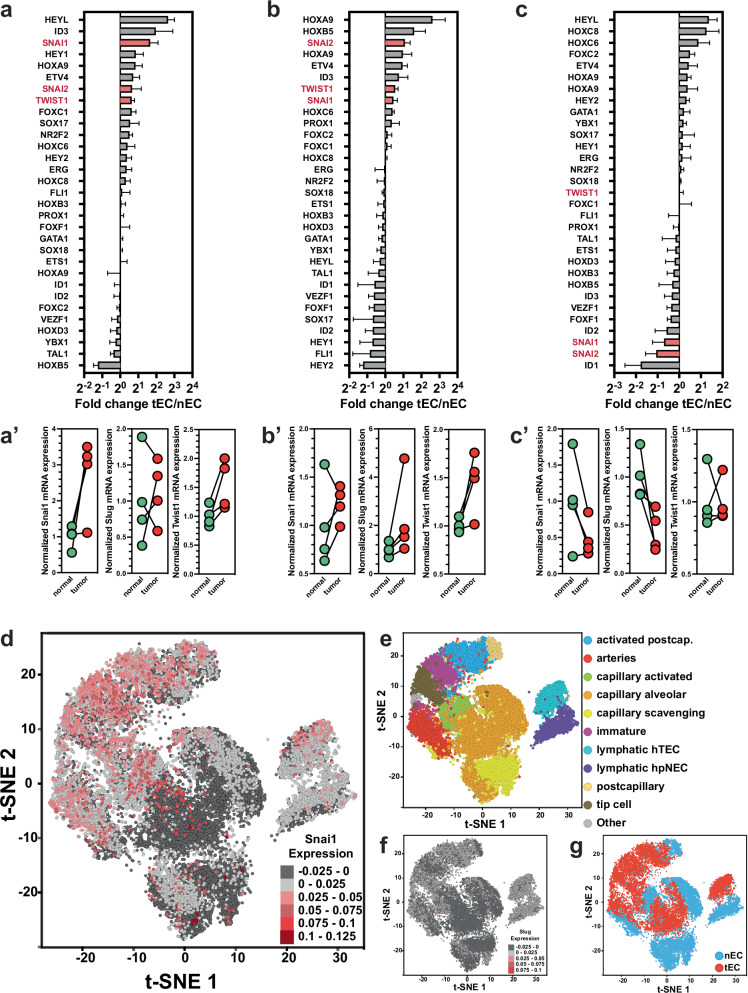
EMT-inducing TFs are upregulated in the tumor endothelium. **a** Expression analysis of TFs involved in vascular development and angiogenesis in tECs from CRC vs. paired nECs samples from GSE77199. a’ Alignment of paired samples from the data set for Snai1, Slug, and Twist1 expression. **b** Expression analysis of TFs involved in vascular development and angiogenesis in tECs from CRC hepatic metastases vs. paired nECs samples from GSE77199. b’ Alignment of paired samples from the data set for Snai1, Slug, and Twist1 expression. **c** Expression analysis of TFs involved in vascular development and angiogenesis in tECs from RCC vs. paired nECs samples from GSE77199. c’ Alignment of paired samples from the data set for Snai1, Slug, and Twist1 expression. **d** Expression analysis of Snai1 in scRNA-seq data (E-MTAB-6308) from lung nECs and tECs. Snai1 expression is primarily elevated in immature ECs, activated postcapillary ECs, and tip cells, EC populations associated with angiogenesis, and largely found in the tEC subset. **e** Overview of cluster analysis of EC subpopulations in the scRNA-seq (E-MTAB-6308) data set. **f** Expression analysis of Slug in scRNA-seq data (E-MTAB-6308) from lung nECs and tECs. Slug is moderately upregulated in tEC vs. nEC, and higher expressed in lymphatic nECs vs. vascular nECs. **g** Overview of tECs and nECs in t-SNE analysis of scRNA-seq data (E-MTAB-6308). Error bars: ±SEM.

Various TFs have been shown to be involved in endothelial activation, wound healing, angiogenesis, and vasculogenesis. We analyzed publicly available RNAseq data sets that allowed for the evaluation of RNA-expression changes in patient-derived tumor endothelial cells (tECs) vs. endothelium in normal tissue (nECs). The EMT-inducing TFs, Snai1, Slug, and Twist1, were upregulated in colorectal cancer (CRC)-associated tECs, both in primary tumors and in hepatic metastases when compared to paired nEC samples from adjacent tissue (Fig. 1a, b) [31]. Within an array of 31 TFs described as being involved in vascular processes, Snai1, Slug, and Twist1 were in both sets at the top of upregulated TFs. Interestingly, in renal cell carcinomas (RCC), tECs Snai1 and Slug were downregulated, and Twist1 expression levels barely changed compared to kidney-derived nECs (Fig. 1c). However, in an additional data set of hepatocellular carcinomas, Snai1, Slug, and Twist1 were again upregulated in tECs compared to normal liver ECs (Supplementary Fig. 1a). For Snai1, expression in tECs could be verified in immunohistologically stained sections of various carcinomas (Supplementary Fig. 1b). Except for skin samples, Snai1^+^-ECs were not found in normal tissue. This included kidney sections where both larger vessels and glomerular capillaries were found negative for Snai1, while the tECs in RCC were strongly positive for Snai1, a finding in conflict with the RCC RNA-expression data. Further insight into the potential role of the TFs came from publicly available scRNA-seq data of pulmonary tECs and nECs (E-MTAB 6308 [32]). High Snai1 expression was primarily found in ECs of the activated postcapillary, immature stalk, and tip cell clusters (Fig. 1d, e). These EC populations are not only part of the tumor microenvironment but also tend to participate actively in angiogenesis. Slug was more uniformly expressed in the various EC subgroups, increased in tECs as compared to nECs, and showed relatively high expression in lymphatic endothelial cells of both normal and tumor tissue (Fig. 1f, g). Twist1 expression was not recorded in the analyzed data set.

### EMT-inducing TFs regulate vascular integrity

**Fig. 2:**
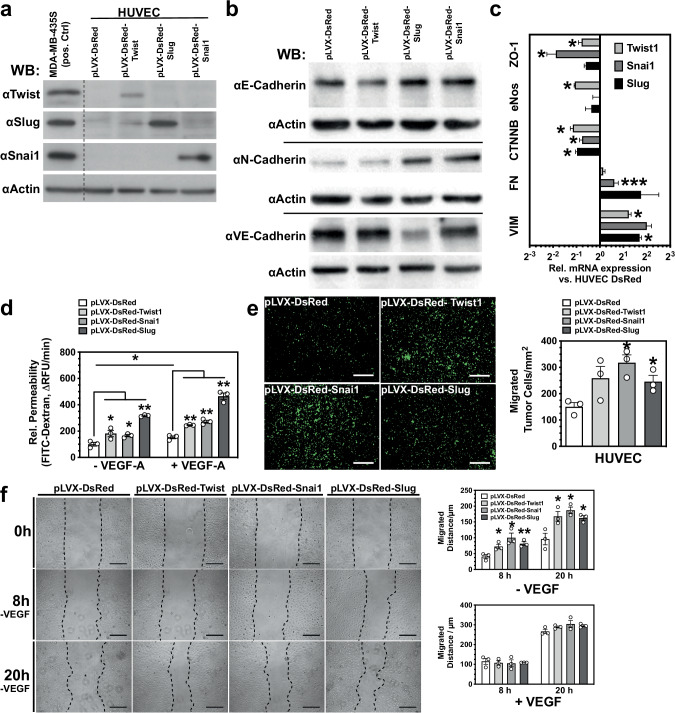
EMT-inducing TFs regulate vascular integrity. **a** Western blot analysis of HUVEC lysate overexpressing Twist, Slug, or Snail1 after lentiviral transduction and puromycin selection. **b** Western blot analysis of cell lysates from HUVEC overexpressing Twist, Slug, or Snail1 for cadherins. **c** RNA expression analysis in HUVEC overexpressing Twist, Slug, or Snail1 demonstrates downregulation of adherents and tight junction proteins (ZO-1 and CTNNB) and upregulation of the mesenchymal markers Vimentin (VIM) and Fibronectin (FN). (*n* = 3). **d** Transwell permeability assay shows increased permeation of high-MW Dextran through EC monolayers. ECs overexpressing Twist, Slug, or Snail1 are more permeable. (*n* = 3). **e** Transmigration of GFP-labeled MDA-MB-231 cells through EC monolayers. Tumor cells migrated faster through ECs overexpressing Twist, Slug, or Snail1. (*n* = 3). **f** Endothelial migration assay (scratch assay). Twist, Slug, or Snail1 overexpression in ECs partially restores reduced migration under VEGF-A depletion (*n* = 3). Scale bars: 100 µm. Error bars: ±SEM. * indicates statistical significance versus control, *p < 0.05, **p < 0.01.

EMT in carcinomas results in phenotypical changes that resemble the alterations occurring in endothelial cells during sprouting angiogenesis: loss of cell-cell contacts, increased motility, and invasiveness. To examine the influence of the main EMT regulating TFs, we generated Human Umbilical Vein Endothelial Cells (HUVEC) that stably overexpressed hSnai1, hSlug, and hTwist by using a lentiviral delivery system (Fig. 2a). Endothelial proliferation was unaffected by overexpression of any of the three EMT-regulating TFs (Supplementary Fig. 2a, b). hTwist suppressed E-cadherin expression, while Slug and Snai1 induced N-cadherin expression (Fig. 2b). Slug and Snai1 also repressed vascular endothelial (VE)-cadherin, an effect not observed after Twist overexpression. This potentially directly affects vascular integrity and patency. mRNA-expression analysis showed upregulation of the mesenchymal markers Vimentin and Fibronectin, but repression of β-catenin (CTNNB) and Zona occludens-1 (ZO-1) after overexpression of Snai1, Slug, or Twist (Fig. 2c).

The role of Twist, Snai1, and Slug in vascular integrity was further confirmed by increased permeability of non-stimulated quiescent EC monolayers for high molecular weight dextran after overexpression of the TFs (Fig. 2d). As a result of the destabilization of tight and adherens junctions (downregulation of VE-cadherin and ZO-1), EC monolayers also were easier penetrable for invasive human breast cancer cells (MDA-MB-231) in a transwell migration assay (Fig. 2e). VEGF-A-deprived ECs still exhibited strong motility when overexpressing either Twist, Snai1, or Slug, while control ECs were dependent on VEGF-A stimulation for motility (Fig. 2f). Thus, overexpression of the TFs was sufficient to induce the hallmarks of angiogenic behavior.

As the three evaluated TFs showed very similar functional effects on endothelial cells, with the difference that Snai1 and Slug also repressed VE-cadherin in vitro, we decided to focus on Snai1 as a target for studies in murine models. This decision was guided by the fact that the analysis of human expression data provided more comprehensive evidence for the upregulation of Snai1 in the tumor endothelium than for Slug and Twist1.

### Tumor growth is enhanced and metastasis suppressed in Snai1^EC-KD^ mice

**Fig. 3:**
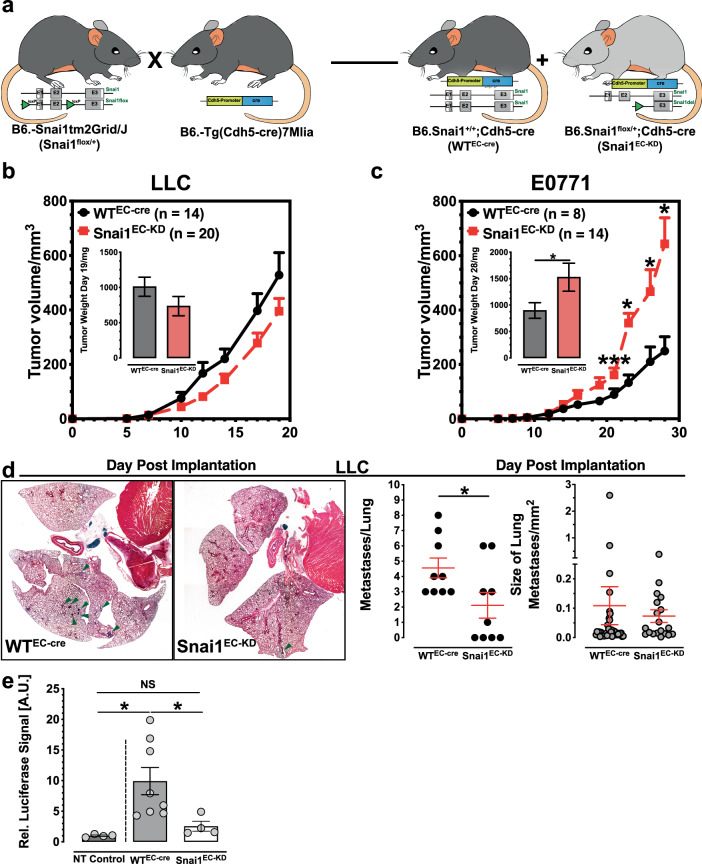
Endothelial-specific inhibition of Snai1 increases tumor growth but reduces metastasis. **a** Breeding scheme generation of mice with endothelial-specific deletion of one Snai1 copy (Snai1^EC-KD^). **b** Growth of LLC tumors is not significantly affected by implantation into Snai1^EC-KD^ mice. **c** Growth rate of E0771 breast carcinoma is increased by implantation into Snai1^EC-KD^ mice. **d** Results of histological evaluation of spontaneous metastasis to the lung in WT^EC-cre^ and Snai1^EC-KD^ mice implanted with LLC tumors. The rate of metastasis is reduced in Snai1^EC-KD^ mice, while the size of individual metastases is not affected. Individual metastases are marked with green arrow heads. *n* = 9. **e** Evaluation of spontaneous metastasis to the lung in WT^EC-cre^ and Snai1^EC-KD^ mice implanted with E0771 tumors. The relative number of seeded tumor cells was evaluated by measuring luciferase activity in homogenized lung tissue (*n* = 4/8, N.T. non-tumor). Error bars: ±SEM. * indicates statistical significance versus control, *p < 0.05, **p < 0.01.

To generate mice with repression of Snai1 expression in the endothelium, we crossed mice carrying one copy of a *Snai1* allele with two loxP sites flanking the first two exons of the gene (B6-*Snai1*^*tm2Grid*^/J) [33] with animals expressing *cre* under the VE-Cadherin promoter (B6-Tg(Cdh5-cre)7Mlia/J) [34]. The cross yielded mice that all expressed *cre* in the endothelium and that either carried one Snai1-loxP allele, which was subsequently deleted by recombination (Snai1^del/+^, henceforth named Snai1^EC-KD^) or that had two copies of the Snai1 WT-allele (henceforth WT^EC-cre^) (Fig. 3a). Because we were interested in studying Snai1 as a potential target for tumor vascular re-engineering in a therapeutic setting, we decided on generating mice heterozygous for Snai1 instead of a complete, homozygous knock-out. Results from mice having the gene expression only partially suppressed represent more closely what might be achievable by a later pharmacological approach to inhibit Snai1. Growth of Lewis lung carcinoma (LLC) was not different after implantation into Snai1^EC-KD^ compared to WT^EC-cre^ mice (Fig. 3b).

However, both E0771 breast carcinomas and B16 melanomas grew significantly faster in the Snai1^EC-KD^ mice (Fig. 3c and Supplementary Fig. 3). Examining lungs from LLC-implanted mice revealed a substantial reduction in the number of metastases in the Snai1^EC-KD^ mice (Fig. 3d). Nevertheless, the size of the individual metastases was similar, indicating that the metastatic colonization and growth at the secondary side were not impaired in Snai1^EC-KD^ mice but that the limiting steps might occur early in the dissemination process. E0771 breast cancer cells also metastasize to the lungs of implanted animals, though at a much slower rate than LLC cells. Thus, macrometastases could not be observed in histological samples of this model, but stably labeling E0771 cells with luciferase by a lentiviral approach (E0771-luc) enabled quantification of disseminated tumor cells in tissue lysates of implanted animals (Fig. 3e). While luciferase activity was strong in the lung lysates from WT^EC-cre^ mice in which E0771-luc tumors were growing, it was not significantly elevated in tumor-bearing Snai1^EC-KD^ mice compared to the lungs of non-implanted control animals.

### Elliptic Fourier analysis enables quantitative assessment of vessel dysfunctionality in archival tissue samples

As previously shown, the regularity of vascular network architecture is a major prerequisite and determinant of corresponding adequate tissue blood perfusion [35]. 3D tissue analysis shows inhomogeneous vessel distribution and irregularly formed individual vessels in tumors. Both correlated with reduced supply. However, in archival 2D tissue slides of patient samples, 3D assessment of vessel architecture is not possible. To be able to compare prospective vascular changes in murine tumor models with the situation in human patients, we explored ways to describe vessel morphology in tissue slides quantitatively. Elliptic Fourier Analysis (EFA) seemed to be a suitable possibility for quantitative morphometric blood vessel analysis. EFA results are independent of the size and orientation of an evaluated shape [36]. As a functional blood vessel shows a circular cross-section to an even, laminar flow, cutting a vessel in an approximately transversal direction results in an equally circular or elliptically shaped section. The shape of transversally cut irregular tumor vessels should diverge from that ideal. To test if EFA also had the necessary discriminatory power to distinguish ideal circular and elliptic vessel sections from the possible divergences observed in defective tumor vessels, we designed five classes of shapes (circular, rectangular, irregular, concave, and indented) and subjected them to EFA (Supplementary Fig. 3a). Indeed, the different shapes yielded different and distinguishable results (in the form of a row of Fourier descriptors (FD), Supplementary Fig. 3b). Moreover, EFA results for the members of the five classes clustered together, demonstrating the possibility of classifying the form of vascular cross-sections according to how they diverge from a circular ideal (Supplementary Fig. 3c). Next, we evaluated the shapes of CD34^+^-stained blood vessels in human breast cancer (BCA) samples. EFA, followed by cluster analysis of the first five FDs, resulted in three distinct classes (Supplementary Fig. 3d). Vessels of normal breast tissue and vessels of several breast carcinoma tissue slides clustered with circular shapes. A second group of tumor blood vessels yielded FDs similar to the indented shapes of the test sets, while a third group presented similarity to rectangular shapes. Interestingly, although these three groups (labeled “normal,” “indented,” and “angular”) represent successive degrees of divergence from an ideal circular phenotype, affiliation with any of the groups was not correlated with vessel density.

### The tumor vasculature in Snai1^EC-KD^ mice exhibits a stabilized phenotype

**Fig. 4:**
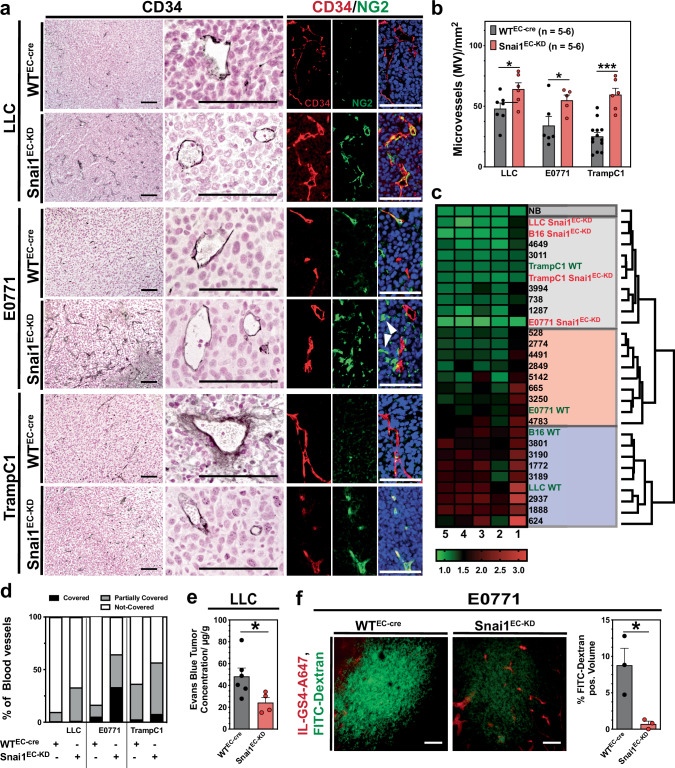
Snai1 inhibition improves vascular maturation. **a** Analysis of blood vessels in sections of various murine tumors grown in WT^EC-cre^ and Snai1^EC-KD^ mice by IHC (vascular marker CD31) and co-IF staining (vascular marker CD34 (red) and pericyte marker NG2 (green)). Tumors grown in Snai1^EC-KD^ mice show generally an increased abundance of perivascular NG2^+^ cells. Additionally, NG2^+^ cells, that are not incorporated into the vessel wall are present in E0771 tumors grown in Snai1^EC-KD^ mice (white arrowheads). **b** Microvessel density (MVD) evaluated in CD31 IHC stained tumor sections. MVD is significantly increased in Snai1^EC-KD^ mice in all tumor models. **c** Results from elliptic Fourier analysis (EFA) of blood vessels in sections of tumors grown in WT^EC-cre^ and Snai1^EC-KD^ mice and a set of human BCA sections. NB normal breast tissue. **d** Evaluation of vessel stabilization by pericyte coverage in sections of various murine tumors grown in WT^EC-cre^ and Snai1^EC-KD^ mice. Sections were co-IF stained for the vascular marker CD34 and the pericyte marker NG2 and evaluated for the degree of coverage indicated by peri-CD34 NG2-staining. **e** Results from Miles assay. Concentration of Evans Blue, calculated from extracts of LLC tumors grown in WT^EC-cre^ or Snai1^EC-KD^ mice. **f** Extravasation of HMW FITC-Dextran in E0771 tumors grown in WT^EC-cre^ or Snai1^EC-KD^ mice. Mice were injected with FITC-labeled HMW-Dextran (FITC-Dextran, green) and Alexa 647-labeled Isolectin GS (IL-GS4-A647, red) to indicated perfused vessels. FITC-Dextran^+^ volume was measured in image z-stacks obtained by CLSM in 150 µm cryo sections. The images show a single plan of the z-stack. Scale bars: 100 µm. Error bars: ±SEM. * indicates statistical significance versus control, *p < 0.05, **p < 0.01, ***p < 0.001.

The vessel density of tumors implanted in Snai1^EC-KD^ mice was strongly increased compared to WT^EC-cre^ tumors (Fig. 4a, b and Supplementary Fig. 4a, b). Examining the morphological characteristics of the individual vessels by EFD demonstrated that only vessels in Tramp C1 tumors (mouse prostate adenocarcinoma) showed already a normal-like vessel shape when implanted in WT^EC-cre^ animals, while other models clustered with the angular and irregular groups. In tumors from Snai1^EC-KD^ mice, a shift toward a normal vessel phenotype was evident in all models (Fig. 4a, c). Pericyte coverage, as a feature of vessel stability, was also increased in tumors grown in Snai1^EC-KD^ mice (Fig. 4a, d). Both the in vitro experiments and the vascular analysis indicated that Snai1 expression interferes with vessel maturation and vascular integrity. To test this in vivo, LLC tumor-bearing mice were injected with Evans blue, a dye with a strong affinity to albumin, forming a complex that can only extravasate in tissues with severely defective endothelium (Miles assay). Evans blue accumulation was strongly reduced in LLC-derived tumors grown in Snai1^EC-KD^ mice as compared to tumors from WT^EC-cre^ animals (Fig. 4e). In E0771 tumors, the effect was even more drastic: extravasation of high molecular weight dextran into the parenchyma was barely detectable in Snai1^EC-KD^ mice, while tumors from WT^EC-cre^ mice showed massive accumulation of the dextran (Fig. 4f).

### Snai1 repression improves drug transport

**Fig. 5:**
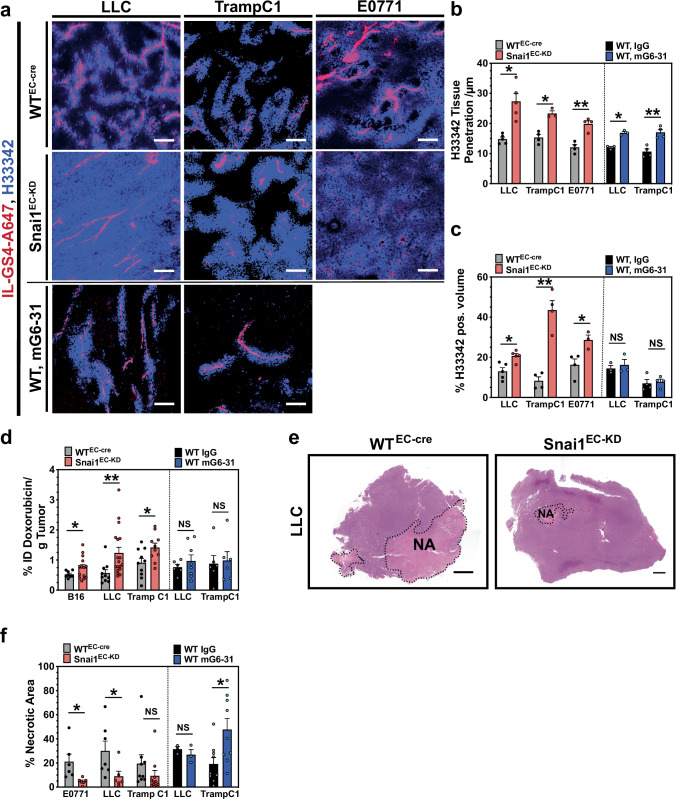
Snai1 inhibition improves vascular integrity and drug transport. **a** Distribution of H33342 in tumor tissue (Z-projection of 30 images, z-step = 1 µm). Cell nuclei reached by the cell permeable nuclear stain H33342 appear blue. Perfused vessel, stained by Alexa fluor 647-labeled isolectin GS4 (IL-GS4-A647) are shown in red. Scale bars: 100 µm. **b** Evaluation of penetration depth of H33342 from the vessel surface into the tumor parenchyma. Both endothelial-specific knock-down of Snai1 and treatment with mG6-31 improved vascular perfusion. (*n* = 3–5). **c** Evaluation of accumulation of H33342 in tumor tissue. Endothelial-specific knock-down of Snai1 improved the accumulation of the drug surrogate H33342. VEGF-A ablation (with mG6-31) did not affect overall accumulation. (*n* = 3–5). **d** Evaluation of accumulation of doxorubicin in tumor tissue shown as the percentage of the injected dose (ID) that was extracted per gram tumor tissue. Endothelial-specific knock-down of Snai1 improved the accumulation of the drug. VEGF-A ablation with mG6-31 did not affect overall accumulation. (*n* = 6–14). **e** H&E stained sections from LLC tumors, grown in WT^EC-cre^ and Snai1^EC-KD^ mice. The necrotic areas in the sections are outlined. Scale bars: 1000 µm. **f** Evaluation of the percentage of necrotic areas observed in central sections from tumors. Endothelial-specific knock-down of Snai1 reduced the amount of necrosis. VEGF-A ablation did not affect necrosis in LLC, but increased necrosis in TrampC1 tumors. (*n* = 6–9). Error bars: ±SEM. * indicates statistical significance versus control, *p < 0.05, **p < 0.01.

Inhibition of Snai1 expression led to a mature, better stabilized, less permeable, and denser tumor vasculature in mouse models. We tested if this normalization of vessel quality and quantity would enhance supply with systemic drugs and compared the results to the effects gained with anti-VEGF therapy using mG6-31, an antibody able to sequester both human and murine VEGF-A [37]. Injection of the DNA-intercalating fluorescent dye Hoechst 33342 (H33342) before sacrificing animals enabled tracking of the distribution of small molecular drugs within the tumor tissue by confocal laser scanning microscopy (CLSM) [38]. In Snai1^EC-KD^ mice, H33342 penetrated significantly deeper into the tumor tissue when measured from the vascular surface (Fig. 5a, b). This effect, indicating an increased supply by individually improved blood vessels, was also observed following anti-angiogenic mG6-31 treatment. However, quantification of the total volume supplied with H33342 showed that only the vascular improvement in Snai1^EC-KD^ mice eventually resulted in a larger proportion of tumors being supplied with detectable amounts of dye, while anti-angiogenic therapy did not significantly affect the total supplied tumor volume (Fig. 5c). As perfused vessel density was strongly reduced after mG6-31 treatment (Supplementary Fig. 4c), both effects of anti-angiogenic treatment—improved individual vessel quality and reduced vessel density—balanced each other out. The same effect was observed for the accumulation of the chemotherapeutic drug doxorubicin (DOX). In all tested models, DOX accumulated at significantly larger amounts in tumors grown in Snai1^EC-KD^ mice compared to tumors from WT^EC-cre^ control mice (Fig. 5d). Again, mG6-31 did not affect DOX accumulation. An overall improvement of supply was also indicated by the reduction of central necrosis in tumors grown in Snai1^EC-KD^ mice (Fig. 5e, f).

### Snai1-KD restricts excessive tip cell formation by activation of Notch signaling

**Fig. 6:**
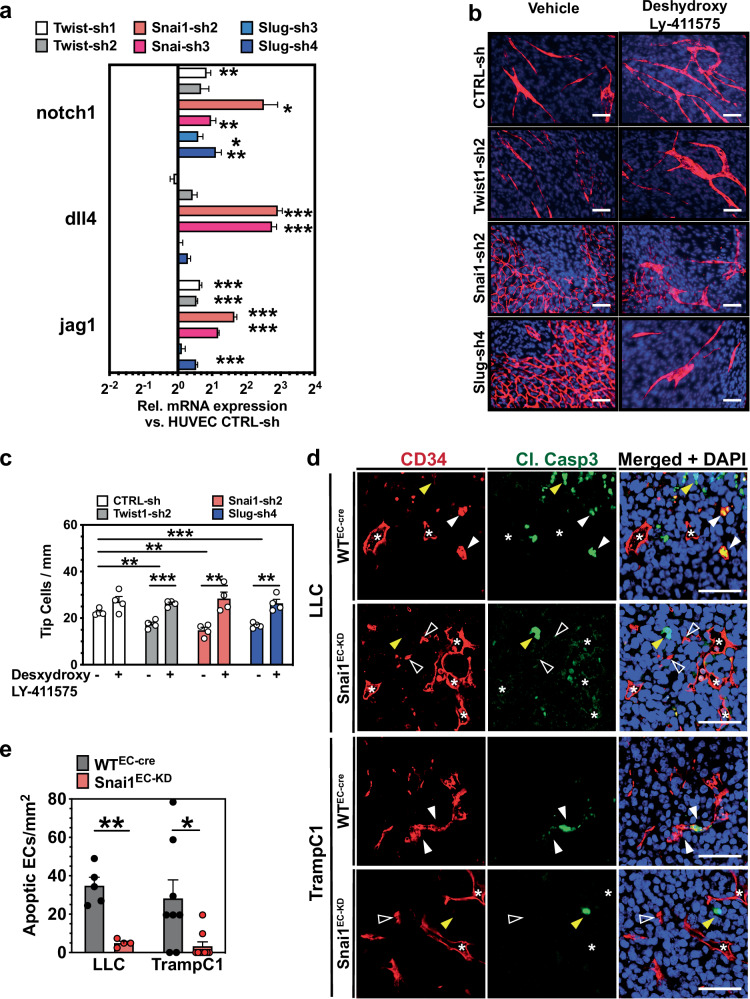
Snai1-KD restricts excessive tip cell formation by activation of Notch signaling. **a** mRNA expression analysis of notch1, dll4, and jag1 in HUVEC after knock-down of either Twist1, Slug or Snai1 via shRNA. (*n* = 4). **b** Endothelial cord formation by HUVEC on NHDF monolayers after knock-down of either Twist1, Slug, or Snai1 via shRNA. Treatment with Deshydroxy LY-411575 rescues the ability of HUVEC to form cord structures impeded by Slug or Snai1 KD. Staining for VE-cadherin (red) and nuclear staining (DAPI, blue). **c** Quantification of tip cells in endothelial cords formed by HUVEC on NHDF monolayers after knock-down of either Twist1, Slug, or Snai1 via shRNA under treatment with Deshydroxy LY-411575. (*n* = 4). **d** Sections from subcutaneously LLC and TrampC1 tumors implanted in WT^EC-cre^ and Snai1^EC-KD^ mice stained for CD34 and the apoptosis marker Cleaved Caspase 3. Luminal non-collapsed vessels (asterisks), collapsed apoptotic vessels (white arrowheads), collapsed non-apoptotic vessels (open arrowheads), and apoptotic tumor cells (yellow arrowheads) are marked. **e** Quantification of apoptotic endothelial cells (CD34^+^/Cl. Casp 3^+^) in sections from subcutaneously LLC and TrampC1 tumors implanted in WT^EC-cre^ and Snai1^EC-KD^ mice. Scale bars: 100 µm. Error bars: ±SEM. * indicates statistical significance with *p < 0.05, **p < 0.01, ***p < 0.001.

Results from the in vitro assays implied an overall pro-angiogenic role of Snai1. However, in all tumor models, MVD was increased in Snai1^EC-KD^ mice, showing the opposite result from what could be expected from the in vitro results. To shed light on the mechanisms underlying this contradiction, we looked into the regulation of the notch pathway by the TFs. Wu et al. have shown that Snai1 negatively regulates Delta-like-4(Dll4)/Notch1 expression in ECs [39], restricting tip cell formation in angiogenic sprouting [40]. Expression profiling in HUVEC revealed that downregulation via shRNA—not only of Snai1 but also of Slug and Twist1—indeed induced expression of both *dll4* and *notch1* mRNA (Fig. 6a and Supplementary Fig. 5a–c). Slug and Twist1 also regulated the notch agonist Jagged1 (Jag1). Network formation of HUVEC in co-culture with fibroblasts was significantly reduced after shRNA-mediated knock-down (Fig. 6b). Interestingly, this was also reflected in a reduction of tip-cell formation (Fig. 6b, c). Treatment of the co-cultures with the γ-secretase inhibitor Deshydroxy LY-411575, which blocks Notch activation [41], partially restored tip-cell formation and network formation in the TF-KD HUVEC. This demonstrated that the anti-angiogenic effects observed in the in vitro assays were notch-mediated.

Thus, we hypothesized that the reduction of excessive sprouting by Snai1 deletion would lead to better stabilized and functional blood vessels, thereby reducing the number of unproductive vessels that would be subsequently pruned and undergo apoptosis. Indeed, tumors in WT^EC-cre^ mice showed a significant proportion of apoptotic endothelial cells, primarily in small, collapsed capillaries (Fig. 6d, e). The extent of endothelial apoptosis was strongly reduced in tumors from Snai1^EC-KD^ mice.

### Snai1 KD reduces hypoxia and invasiveness and improves response to chemotherapy

**Fig. 7:**
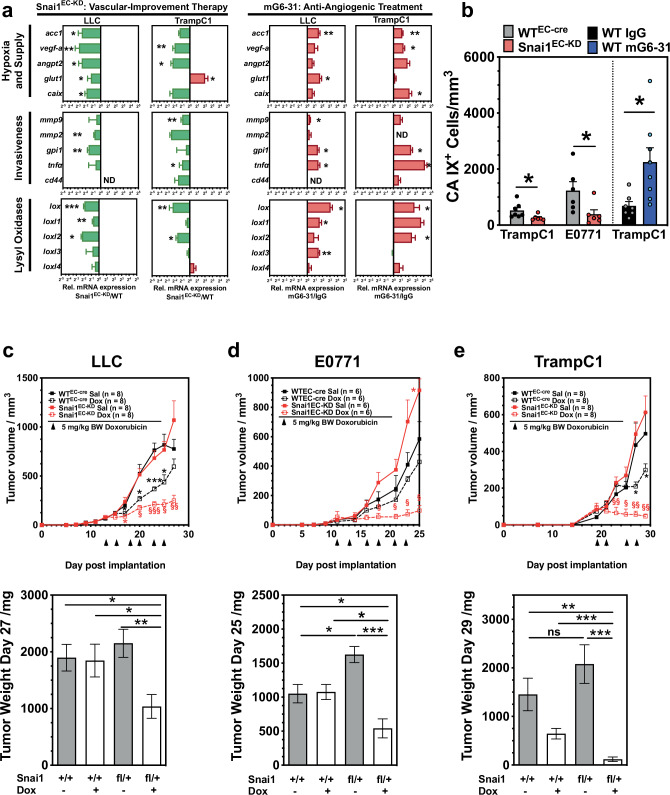
Snai1 inhibition improves the efficacy of chemotherapeutic treatment. **a** Comparative mRNA expression analysis of genes related to hypoxia/nutritional supply, or invasiveness and of lysyl oxidases in tumors grown in Snai1^EC-KD^ and of tumors after anti-angiogenic therapy with mG6-31. Anti-angiogenic therapy leads to a hypoxic, highly condensed, and more aggressive phenotype, while the improved tumor vasculature in Snai1^EC-KD^ animals reduces hypoxia and malignancy. (*n* = 4). **b** Evaluation of CA-IX IHC positive staining in sections of LLC, E0771, and TrampC1 tumors. **c** Response of LLC tumors implanted in WT^EC-cre^ and Snai1^EC-KD^ mice to DOX. Growth curve (days when DOX was applied by i.p. injection are indicated), and weight of tumors at day of sacrifice (d27 post implantation). **d** Response of E0771 mammary carcinoma implanted in WT^EC-cre^ and Snai1^EC-KD^ mice to DOX. Growth curve (days when DOX was applied by i.p. injection are indicated), and weight of tumors at day of sacrifice (d25 post implantation). **e** Response of TrampC1 prostate adenocarcinoma implanted in WT^EC-cre^ and Snai1^EC-KD^ mice to DOX. Growth curve (days when DOX was applied by i.p. injection are indicated), and weight of tumors at day of sacrifice (d29 post implantation). Scale bars: 100 µm. Error bars: ±SEM. * indicates statistical significance versus control, ^§^ indicates statistical significance in treated Snai1^EC-KD^ groups versus all three control groups, *,^§^*p* < 0.05, **, ^§§^*p* < 0.01, ***, ^§§§^*p* < 0.001.

Endothelial Snai1-KD improved vascular stabilization, increased MVD, and better drug delivery into implanted tumors. This suggested an overall improved drug supply to tumors. We evaluated mRNA expression of several indicators of supply, oxygenation, invasiveness, and metastasis. In tumors from Snai1^EC-KD^ mice, expression of hypoxia markers (*vegf-a*, *angpt2*, *glut1*, *caix*) was generally reduced (Fig. 7a). The same was observed for mmp2 and mmp9, metalloproteases connected to tumor cell invasion, for glucose-phosphate isomerase 1 (*gpi1*), *tnfα*, lysyl oxidases (*lox, loxl1-4*), and the progenitor marker *cd44* that is also linked to increased metastasis. Most of these genes are hypoxia-regulated and induced via Hif1α. Thus, we tested also for expression of acetyl-coenzyme A carboxylase (acc1), an indicator of metabolic stress that is not hypoxia-regulated. *Acc1* levels were also significantly reduced in Snai1^EC-KD^-derived tumors. In contrast, the same tumors (LLC and TrampC1) showed an overall increase in the expression of hypoxia markers, lysyl oxidases, and invasion-related genes after mG6-31 treatment. Results from the mRNA profiling experiments were confirmed by IHC staining for CA-IX (Fig. 7b).

To test if the observed improvement in supply and drug transport also resulted in an increased response to therapy, we treated tumor-bearing WT^EC-cre^ and Snai1^EC-KD^ mice with chemotherapy. Three different tumor models (LLC, E0771, and TrampC1) were treated with DOX (5 mg/kg B.W.) after implanted tumors were fully established (Fig. 7c–e). In all models, tumors in Snai1^EC-KD^ mice reacted significantly stronger to therapy. E0771 tumors did not respond to DOX with an observable reduction of tumor growth when implanted in WT^EC-cre^ mice. However, although the untreated E0771 tumors grew faster in the Snai1EC^-KD^ mice, they also reacted with significant growth reduction to DOX in this background, proving that vascular improvement via Snai1 targeting can confer sensitivity toward an otherwise ineffective therapy.

## Discussion

The dysfunctional tumor vasculature not only hampers drug delivery and distribution but also leads to chronic hypoxia and nutritional undersupply in cancer. This, in turn, reduces the effectiveness of radiotherapy and promotes tumor progression by increasing invasiveness and metastasis. A potential strategic approach to “repair” the tumor vasculature with the explicit aim of improving response to therapy and reducing adverse toxicity on non-malignant tissue has to take the vascular network in its entirety into account. The dysfunctionality of the tumor vasculature manifests itself on three different levels: (1) impaired stabilization and patency of the individual capillaries, (2) reduced density and heterogeneous distribution of vessels, and (3) morphological changes of the vessels resulting in irregular, non-laminar flow and increased thrombosis [35, 42, 43]. Endothelial Snai1 inhibition improved the tumor vasculature, with respect to all three parameters—a stark contrast to anti-VEGFA therapy that only improves vessel maturation but often reduces the already critical vessel density even further. A challenge is the quantitative assessment of the morphological changes. Elliptic Fourier analysis has been successfully used for shape classification in a number of diagnostic settings, e.g., in the evaluation of non-melanoma skin lesions, the analysis of nuclei shapes in myeloproliferative diseases, or the tracking of changes in collagen spindle shapes in breast cancer progression [44–46]. It has also been employed in vessel analysis, namely the post-surgery follow-up in aortic remodeling [47, 48]. In this study, we have used elliptic Fourier analysis to evaluate and classify microvessels in the tumor microenvironment. The method not only allowed for a fast and reliable assessment of vascular defects in archival tissue sections but also revealed changes in the tumor vasculature in Snai1^EC-KD^ animals that indicated progress toward a phenotype of vessels in normal tissue.

It was previously shown that Snai1 is essential for TGFβ-induced EndMT but not sufficient to induce EndMT by itself [24]. We found that Twist1 and Slug overexpression also did not induce a full EndMT. However, changes in E/N-cadherin and β-catenin expression indicated the acquisition of a partial mesenchymal phenotype. Thus, while Zeisberg et al. showed that cancer-associated fibroblasts (CAFs) can be derived from local ECs in a Snai1-dependent manner by TGFβ stimulation [23], most ECs in the tumor microenvironment do not commit fully to a mesenchymal fate. Mesenchymal characteristics are only acquired to the extent necessary for invasive sprouting angiogenesis. Our data also shows that this endothelial “mesenchymalization” is persistent under the constant pro-angiogenic stimulus within neoplasia. The alterations we observed in ECs upon overexpression of the EMT-regulating TFs also explain the vascular defects observed in many malignant tumors, increased vascular permeability, poorly controlled instigation of novel sprouts, and vascular destabilization. We showed that Twist1 and Snai1/Slug often regulated different proteins with complementary functions. While Twist1 represses β-catenin, Snai1, and Slug inhibit VE-cadherin expression. Both VE-cadherin and β-catenin are necessary for endothelial barrier function, and their suppression increases permeability [49–51]. The differences in regulating complementary proteins in ECs might be explained by findings that indicate that Snai1 is more an inducer of EMT, while Twist1 is more important for maintaining a mesenchymal state [52]. However, most work on Twist1, Snai1, and Slug has been conducted in epithelial cells. Details on their often matching or complementary roles in endothelial cells and in angiogenesis need to be explored.

Vessels in Snai1^EC-KD^ mice-derived tumors were not only better stabilized but also more abundant. It has previously been shown that DLL4/Notch1 inhibition increases vascularization, leading to unproductive angiogenesis that inhibits tumor growth [40, 53, 54]. Repression of endothelial Snai1-expression in the strongly pro-angiogenic tumor microenvironment has the opposite effect, reducing the ill-fated excessive sprouting via increased Notch1/Dll4 expression, leading to stable vessels that are no longer prone to constant remodeling and degradation. Thus, while Snai1^EC-KD^ is not pro-angiogenic in terms of inducing vessel formation, it can result in an increased vessel density by contributing to a mature neo-vessel phenotype that is less prone to pruning.

Reduced vascular permeability is one factor contributing to the decreased rate of metastasis observed in Snai1^EC-KD^ mice. A second major factor is the improved supply and better oxygenation of the implanted tumors, which significantly reduces invasiveness. Hypoxia markers were significantly downregulated in tumors grown in Snai1^EC-KD^ mice. Hypoxia is a primary driving force for invasive behavior [55–58]. Several Hif-1α target genes, like glucose-6-phosphate isomerase, MMPs, lysyl oxidases, cathepsins, and plod2, contribute to invasiveness and metastasis by increasing cell motility and ECM remodeling [57–60]. We demonstrated that the expression of these metastasis promoters is also positively affected after endothelial Snai1 targeting. In contrast, anti-angiogenic therapy effectively reduces overall supply within tumors, leading to upregulation and stronger expression of genes that regulate invasiveness and metastasis. Furthermore, these results underline that anti-angiogenic drugs foremost have the effects they were developed for: decrease vascularization, reduce supply, and consequently starving the tumor and thereby restricting its growth.

Although the beneficial effect of reduced endothelial Snai1 expression on supply, metastasis, and drug response was evident in all tumor models, we also observed differences, especially in the effect on primary tumor growth. While E0771 and B16 tumors grew faster in Snai1^EC-KD^, the growth of LLC tumors was not affected. A possible reason behind the different sensitivity is that some murine tumors are growth-limited by their inadequate and defective vasculature [7]. Those tumors react strongly to additional supply restriction by anti-angiogenic therapy and might, on the other hand, react with increased growth to a normalization of their vasculature. Tumors that are restricted in their growth by other mechanisms react less sensitive to vascular changes.

Targeting TFs is not trivial. However, some work has shown the principal feasibility of such an undertaking, either by preventing TF-dimerization, blocking their binding to regulatory DNA elements, or by inhibiting activating modulators [61–64]. This study, like others that have demonstrated the critical involvement of TFs and other difficult-to-target proteins, shows the necessity to broaden therapeutic approaches to include undruggable targets. Especially in the context of complex diseases like cancer, drugging these key mediators, often located far down in the intracellular signaling pathways, has the prospect of causing precisely focused but nevertheless powerful effects.

## Material and methods

### General

If not otherwise indicated, chemicals were purchased from Sigma-Aldrich (Munich, Germany) or Carl Roth (Karlsruhe, Germany). Protein concentrations were determined with the Pierce BCA Kit (Thermo Fisher, Rockford, IL), using a 30 min incubation time at 60 °C.

#### Cell culture

LLC (ATCC Cat# CRL-1642, RRID:CVCL_4358) TrampC1 (ATCC Cat# CRL-2730, RRID:CVCL_3614) B16-F10 (ATCC Cat# CRL-6475, RRID:CVCL_0159), MDA-MB-231 (ATCC Cat# HTB-26, RRID:CVCL_0062) and HEK293T (ATCC Cat# CRL-3216, RRID:CVCL_0063) cells were obtained from ATCC (Manassas, VA). E0771 (Cat# 940001-A, eq. RRID:CVCL_B0A2) cells have been purchased from Tebu-Bio (Offenbach, Germany). All tumor cells were maintained in DMEM (Gibco) with 10% FBS and Penicillin/Streptomycin at 37 °C, 5% CO_2_. Cell lines were tested for mycoplasma contamination every 6–8 weeks and found negative for contamination.

The human breast cancer cell line MDA-MB-231 cells were authenticated by the cell line authentification service of the German Collection of Microorganisms and Cell Cultures (DSMZ, Braunschweig, Germany).

HUVEC-2 were purchased from B.D. Biosciences (Franklin Lakes, NJ) and maintained in EGM-2 media (Lonza, Basel, Switzerland). NHDF were purchased from Lonza and cultivated in DMEM (Gibco) with 10% FBS, penicillin/streptomycin, 1% non-essential amino acid supplement (ThermoFisher), and ascorbic acid-2-phosphate (SigmaAldrich) at 37 °C, 5% CO_2_.

### Production of lentiviral particles and generation of HUVEC expressing hSnai1, hSlug, and hTwist

The entire CDS of hSnai1, hSlug, and hTwist CDS was amplified from HUVEC cDNA. The amplified DNA was cloned behind the IRES sequence into the lentiviral vector pLVX-DsRed-IRES-puro (Clontech, Mountain View, CA). Lentiviral particles were generated in HEK 293T cells by co-transfection with the pCMV-dR8.9 (RRID:Addgene_8454) and pCMV-VSV-G (RRID:Addgene_8454) [65] (both plasmids were obtained from Addgene, Cambridge, MA), using a standard CaCl_2_-based transfection method. The supernatant was used to transfect HUVEC-2. Stable cells selected with puromycin (0.5 µg/mL). To generate a control cell line, HUVEC-2 cells were transfected with lentiviral particles produced in HEK 293T cells using the pLVX-DsRed-IRES-puro plasmid.

For silencing sets with 4 (hSlug, hTwist1) or 5 (hSnai1) shRNAs cloned in the lentiviral expression vector pGIPZ (RRID:Addgene_121488) were purchased from Open Biosystems (Huntsville, AL, meanwhile available through Horizon Discovery). A control pGIPZ with a non-silencing shRNA was used. Production of lentiviral particles, transfection, and selection were performed as described above for pLVX-based O.E. vectors.

### Generation of Snai1^EC-KD^ mice

B6.Cg-Tg(Cdh5-cre)7Mlia/J (B6.Cdh5-Cre) [34] and B6;129S-*Snai1*^*tm2Grid*^/J (Snai1-loxP, (RRID:IMSR_JAX:010686) [33]) mice were obtained from Jackson Laboratories (Bar Harbor, Maine). Both lines were backcrossed into C57Bl/6J (RRID:IMSR_JAX:000664) for ten additional generations in house. A pure C57Bl/6J background was verified by genome scanning. Heterozygous B6.Snai1-loxP animals were generated and used for crosses with B6.Cdh5-Cre mice. The cross yielded a 50/50 mendelian ratio of animals that carried one copy of the Snai1-loxP allele and the Cdh5-cre allele (Snai1^EC-KD^) and animals that did not carry the Snai1 mutant allele but expressed *cre* in the endothelium (WT^EC-cre^), which were used as control mice in the respective studies.

### Tumor models and treatment

#### Tumor engraftment

TrampC1 (5 × 10^6^ cells in PBS) and LLC (1 × 10^6^ cells in Matrigel) tumors were generated by subcutaneous injection in the dorsal region of male mice, B16 melanomas (1 × 10^6^ cells in PBS) by subcutaneous injection in the dorsal region of female mice. E0771 (1 × 10^6^ cells in PBS) breast adenocarcinomas were generated by injection of cells into the inguinal mammary fat pad of female mice. Group sizes were calculated to detect a projected effect size with a probability of >95% according to standard statistical procedures [66].

Animals in the individual experiments were mattes from several litters and age- (8–12 weeks, in individual experiments) and sex-matched. For each experiment, tumor-bearing mice were randomly assigned to the different treatment groups just prior to the start of treatment. In treatment studies where tumor growth was a critical endpoint, assessment of tumor size was performed blinded by a second researcher.

#### Exclusion of data

Animals that never developed tumors due to a take rate lower than 100% were excluded from the studies. All data from animals that died or had to be sacrificed prior to the scheduled termination of the experiment was excluded.

#### Tumor treatment

Doxorubicin (5 mg/kg B.W.) was administered by intraperitoneal injection on indicated days.

Tumor growth was followed by measuring perpendicular diameters of the tumors with a vernier caliper. Tumor volume was calculated using the equation *V* = *π*/6 × *l* × *w*^2^. In addition, tumors were excised post-mortem and weighted. Only tumors that could be excised completely without additional invaded tissue were used for weight measurements.

H&E, IHC, and IF stainings were performed using standard techniques on formalin-fixed paraffin-embedded sections. Tissues for quantitative evaluation were processed in parallel. For quantification, whole H&E or IHC-stained tissue sections were imaged on a Keyence BZ-X800 (Keyence, Osaka, Japan) microscope equipped with an automated stage. The whole virtual slide was used for quantification using the ImageJ software package (rsbweb.nih.gov/ij/). Tumor sections stained by immunofluorescence were acquired on a Nikon A2 laser scanning confocal microscope equipped with 20x and 60x objectives (Nikon, Tokyo, Japan). Lower magnification images were acquired for quantification to ensure coverage of larger, more representative fields. Additionally, higher magnification images were acquired within the same area for illustration.

Antibodies used for IHC/IF or WB: NG2 (Millipore Cat# AB5320, RRID:AB_11213678) Cleaved Caspase-3 ((Cell Signaling Technology Cat# 9661 (also NYUIHC-314, 9661S, 9661L), RRID:AB_2341188), Carbonic Anhydrase IX (Santa Cruz Biotechnology Cat# sc-25599, RRID:AB_2066539), CD31 ((Santa Cruz Biotechnology Cat# sc-28188, RRID:AB_2267979), CD34 (Abcam Cat# ab8158, RRID:AB_306316), Collagen IV (Bio-Rad Cat# 2150-1470, RRID:AB_2082660), Ki67 (Abcam, ab16667), β-Actin (Santa Cruz Biotechnology Cat# sc-1615, RRID:AB_630835).

### Endothelial permeability

A total of 8000 HUVEC-2 were seeded in 200 µL EGM-2 media in the upper compartment of 24-well transwell inserts (0.3 µm pores size, Corning, New York, NY). The lower compartment was supplied with 500 µL EGM-2. Cells were allowed to proliferate and form a confluent monolayer for 4 days before being FBS and serum starved for 6 h in EBM-2 basal media. The lower compartment was then filled with 800 µL EBM-2 (±20 ng/mL VEGF-A) supplemented with 250 ng/mL FITC-Dextran (40 kDa, Sigma-Aldrich), the upper compartment was filled with 300 µL (±20 ng/mL VEGF-A). Diffusion of the fluorescent dextran into the upper compartment was measured for 5 h in an automated plate reader (Victor3, PerkinElmer).

To quantify transendothelial tumor cell migration of tumor cells, 15,000 HUVEC were seeded in 200 µL EGM-2 media in the upper compartment of 24-well transwell inserts with 3 µm pore size. The lower compartment was supplied with 500 µL EGM-2. Cells were allowed to proliferate and form a confluent monolayer for 3 days before being FBS and serum starved for 6 h in EBM-2 basal media. The lower compartment was then filled with 800 µL DMEM with 10% FBS. A total of 1.5 × 10^5^ MDA-MB-231 cells stably expressing GFP were added in 50 µL DMEM w/o FBS into the upper chamber. After 4 h, membranes were removed from transwell inserts and mounted. Transmigrated tumor cells were counted under an epi-fluorescence microscope.

### 2D vessel shape analysis by EFA

Human tumor samples were stained for CD34 (B.D. Bioscience) using a standard IHC protocol. High-power images were acquired on a Keyence BZ-9000 microscope with a 40 x Nikon apoplan lens and merged together using the Keyence Analyzer II software to obtain images that covered the whole tumor section. Analysis was performed using the elliptic Fourier descriptor plugin (EFD, part of the Fourier shape analysis tool set, https://imagejdocu.list.lu/plugin/analysis/fourier_shape_analysis/start) in ImageJ (RRID:SCR_003070) [67]: the transversally cut vessel was marked using the freehand selection tool and the EFD plugin was run to collect the first five Fourier descriptors. Average values obtained from all vessels of individual tumor samples were compared using Cluster 3.0 (http://bonsai.hgc.jp/~mdehoon/software/cluster/software.htm) (Euclidian Distance; Average Linkage) and visualized using Java TreeView 1.2.0 (http://jtreeview.sourceforge.net/) [68].

### Analysis of publicly available scRNA-seq data

The batch-corrected normalized data set was downloaded from https://endotheliomics.shinyapps.io/lung_ectax/ and analyzed using Orange 3.35 (RRID:SCR_019811, https://orangedatamining.com) for expression of Snai1 and Slug (The data are also available at https://www.ebi.ac.uk/biostudies/arrayexpress/studies/E-MTAB-6308).

### Hoechst distribution, lectin vessel staining, and 3D image evaluation

To monitor the intra-tumoral distribution of drugs, 50 µL of Hoechst 33342 stock solution (Sigma, 20 mg/mL in 0.9% NaCl) and 50 µL of Alexa 647-labeled Isolectin GS-B4 (Life Technologies, Darmstadt, Germany. A total of 500 µg/mL in 0.9% NaCl) were injected i.v. into tumor-bearing mice 20 min before sacrificing the animal. Tumors were removed and flash-frozen in OCT (Sakura Finetek, Torrance, CA).

For Hoechst 33342 tissue penetration and 3D-vessel evaluation, tissue was cut on a cryotome to 200 µm slices and mounted on glass slides. Z-stacks were acquired by confocal (Nikon A2, 20x objective) imaging by excitation with a 405 nm and 647 nm laser line. Tissue penetration was measured as the maximal distance from the vessel surface (by Alexa 647 staining) that Hoechst 33342 staining was present using ImageJ. For this purpose, the acquired z-stacks were evaluated at the same tissue depth for isolated, longitudinal cut blood vessels. The maximal distance of Hoechst 33342 staining was measured perpendicular to both sides of each blood vessel, and the arithmetic mean of the two values was used. Each blood vessel was evaluated at several positions. At least 10 vessels per stack and four stacks per biological sample were evaluated.

### Evans Blue extraction (Miles assay)

Animals were injected 30 min prior to euthanasia with 2% Evans Blue in 0.9% NaCl (2.5 mL/kg B.W.). After sacrifice, animals were perfused through the left ventricle with 10 mL PBS. From each tumor, three non-necrotic samples of 100–200 mg were taken from different regions and homogenized with a turrax in 9 volumes of formamide. The homogenized samples were incubated at 40 °C for 36 h and cleared by centrifugation. The absorbance of the cleared supernatant was read at 620 nm and 740 nm. The absorbance corrected for hemoglobin contamination was calculated according to A_620_ (corrected) = A_620_ − A_740_ and compared to a standard curve to calculate the relative amount of extravasated Evans Blue. Average values of the three samples per tumor were used as an independent biological sample.

### Biodistribution of doxorubicin

For biodistribution studies, a bolus of 100 µg doxorubicin in 0.9% NaCl was injected intraperitoneally on specified days to doxorubicin naïve animals. Mice were sacrificed 2 h post-injection when doxorubicin could be expected to be cleared from the bloodstream [69]. Tissue samples were flash frozen and stored at –80 °C until extraction. The method described by Laginha et al. was used with slight modifications [70]. In brief: Tissue samples were homogenized by sonification in 9 parts (v/w) water. A total of 200 µL homogenate was combined with 50 µL 10% Triton X-100 (v/v) and 750 µL 0.75 N HCl in 2-propanol. The mixture was vortexed briefly and extracted for 12 h at −20 °C. Samples were again vortexed at r.t. and cleared by centrifugation (20 min, 4 °C, 20,000 × *g*). Fluorescence was read (Ex.: 470 nm, Em.: 590 nm) in a microplate reader and corrected against extracts from tissue samples of non-treated animals. A standard curve was established by adding defined amounts of doxorubicin/Doxil to homogenates of non-treated tissue samples prior to extraction.

### RNA-isolation

RNA was isolated from experimental murine tumors using the RNeasy Kit (Qiagen, Hilden, Germany) according to the manufacturer’s recommendations.

### mRNA-Expression analysis

mRNA-expression levels were quantified using the GeXP-System (Beckman Coulter, Krefeld, Germany). Protocols for reverse transcription, amplification, labeling, gel electrophoresis, and quantification were used as recommended by the manufacturer. RNA levels were normalized to levels of housekeeping genes β-2-microglobulin (*b2m*) and ribosomal protein S29 (*rps29*) in murine, b2m, and ribosomal protein S13 (rps13) in human samples, respectively [71]. Analysis was done with three technical replicates per biological sample. Mean values of technical replicates were used for statistical analysis.

### Validation of Snai1-knockdown in Snai1^EC-KD^ blood vessels

LLC Tumors were implanted in Snai1^EC-KD^ and WT^EC-cre^ mice and grown for 16 days. Immediately after sacrificing animals, tumors were excised and cut into small blocks of approximately 5 mm in length and depth. The blocks were submerged in OCT medium (Cat# 4583, Sakura, Torrance, CA) in standard embedding forms and immediately snap-frozen in liquid nitrogen. From this point on, the samples were stored and handled at −80 °C or on dry ice). The tissue was cut into 15 µm thick sections on a cryotome (CM3050, Leica, Wetzlar, Germany), the sections were transferred to PPS-membrane frame slides (Cat# 11600294, Leica) and again stored at −80 °C. A shortened IHC staining procedure was established to reduce RNA degradation: the CD34 antibody (Abcam Cat# ab8158, RRID:AB_306316) was diluted 1:50 in antibody diluents (S080983-2, Agilent, Santa Clara, CA) and applied to the section for 90 min at r.t. The sections were washed three times with PBS for 5 min and stained using a polymer HRP kit (ImmPRESS HRP Goat anti-rat, Cat# MP-7404-50, Vector Laboratories, Newark, CA) according to the manufacturer recommendations but with a shortened incubation time of 60 min. Sections were counterstained with hematoxylin.

On a laser capture microdissection microscope (LMD 6500, Leica), a technique that allows for the isolation of specific cells or tissues under direct microscopic visualization, 40–70 CD34^+^ blood vessels per biological sample were dissected and pooled. To assess RNA expression in non-vessel fractions of the tumor, 20–30 small sections were dissected from the tissue in parallel, avoiding CD34^+^ areas.

RNA was isolated from the microdissected sections using the PicoPure RNA Isolation Kit (Cat# KIT0202, Arcturus, Mountain View, CA) and amplified with the RiboAmp HS Plus cDNA kit (Cat# KIT0529, Arcturus). The resulting cDNA was used to quantify Snai1 mRNA expression using the methods described above.

### Statistical analysis

Statistical analyses were done using the Prism5 Software (RRID:SCR_002798, GraphPad, La Jolla, CA). Differences between the two groups were analyzed using an unpaired, two-tailed Student’s *t*-test. In parallel, the samples were tested for significant variation of variance, and if necessary, a Welch correction was included in the statistical analysis. For statistical analysis of metastatic incidence and size of metastases between groups, the Mann–Whitney test was used as a Gaussian distribution could not be assumed. All statistical tests were performed between sets of individual biological replicates.

### Supplementary information

Supplemental data

## Data Availability

Data analyzed in this study were obtained from Gene Expression Omnibus (GEO (RRID:SCR_005012), (https://www.ncbi.nlm.nih.gov/geo/; GSE 51401 and GSE77199 bulk tEC vs. nEC data) and from Array Express (https://www.ebi.ac.uk/biostudies/arrayexpress/ E-MTAB-6308 scRNA-seq tEC and nEC data).
