# Supplementary material for: Normalization of Snai1-mediated vessel dysfunction increases drug response in cancer
Source: Oncogene. 2024 Aug 2;43(35):2661–76. doi: 10.1038/s41388-024-03113-1 (PMC11347376; doi:10.1038/s41388-024-03113-1)
Supplement: Supplementary file 1 — Supplemental data [file 41388_2024_3113_MOESM1_ESM.pdf]

# Normalization of Snai1-mediated vessel dysfunction increases drug response in cancer

Helene Hoffmann[1,2#], Martin Wartenberg[3,4,#], Sandra Vorlova[5], Franziska Karl-Schöller[1], Matthias Kallius[1,2], Oliver Reinhardt[1], Asli Öztürk[1], Leah S. Schuhmair[1], Verena Burkhardt[1], Sabine Gätzner[6], Daniela Scheld[7], Rajender Nandigama[1], Alma Zernecke[5], Sabine Herterich[7], Süleyman Ergün[1], Andreas Rosenwald[3], and Erik Henke[1,2*]

## - Supplemental Data -

[1] Institute of Anatomy and Cell Biology, Universität Würzburg, Koellikerstrasse 6, Würzburg 97070, Germany

[2] Graduate School of Life Science, Universität Würzburg, Josef-Schneider-Strasse 2, 97082 Würzburg, Germany

[3] Institute of Pathology, Universität Würzburg, and Comprehensive Cancer Center Mainfranken (CCCMF), Josef-Schneider-Strasse 2, 97082 Würzburg, Germany

[4] Institute of Tissue Medicine and Pathology (ITMP), Universität Bern, Murtenstrasse 31, 3008 Bern, Switzerland

[5] Institute of Experimental Biomedicine II, Universitätsklinikum Würzburg, Josef-Schneider-Strasse 2/D16, 97082 Würzburg, Germany

[6] Chair Tissue Engineering and Regenerative Medicine (TERM), Universitätsklinikum Würzburg, Roentgenring 11, Würzburg 97070, Germany

[7] Zentrallabor, Universitätsklinikum Würzburg, Josef-Schneider-Strasse 2, 97082 Würzburg, Germany

[#] These authors contributed equally to this work

* To whom correspondence should be addressed:

Erik Henke

Institute for Anatomy and Cell Biology

Universität Würzburg.

Koellikerstrasse 6

97070 Würzburg, Germany

Email: erik.henke@uni-wuerzburg.de

Tel: +49-(0)931-3183270, Fax: +49-(0)931-329363

**Supplemental Figure 1:**

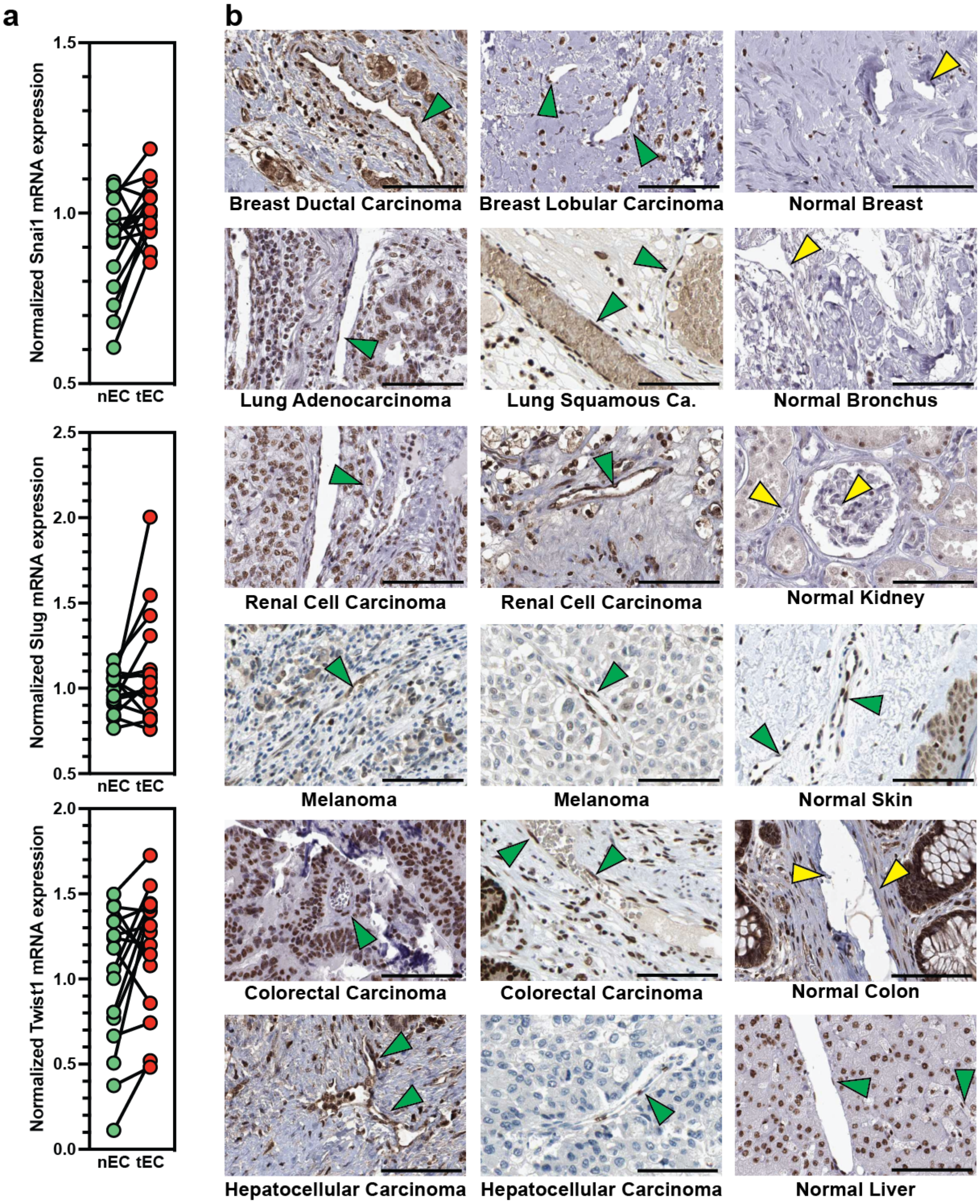


**(a)** Alignment of paired samples from the GSE 51401 data set for Snai1, Slug, and Twist1 expression (https://www.ncbi.nlm.nih.gov/geo/query/acc.cgi?acc=GSE51401).

**(b)** IHC staining for Snai1 in human tumor samples and in corresponding normal tissue (https://www.proteinatlas.org/ENSG00000124216-SNAI1/pathology). Green arrowheads show Snai1+ ECs and yellow arrowheads show Snai1- ECs (in normal tissue).

**Supplemental Figure 2**

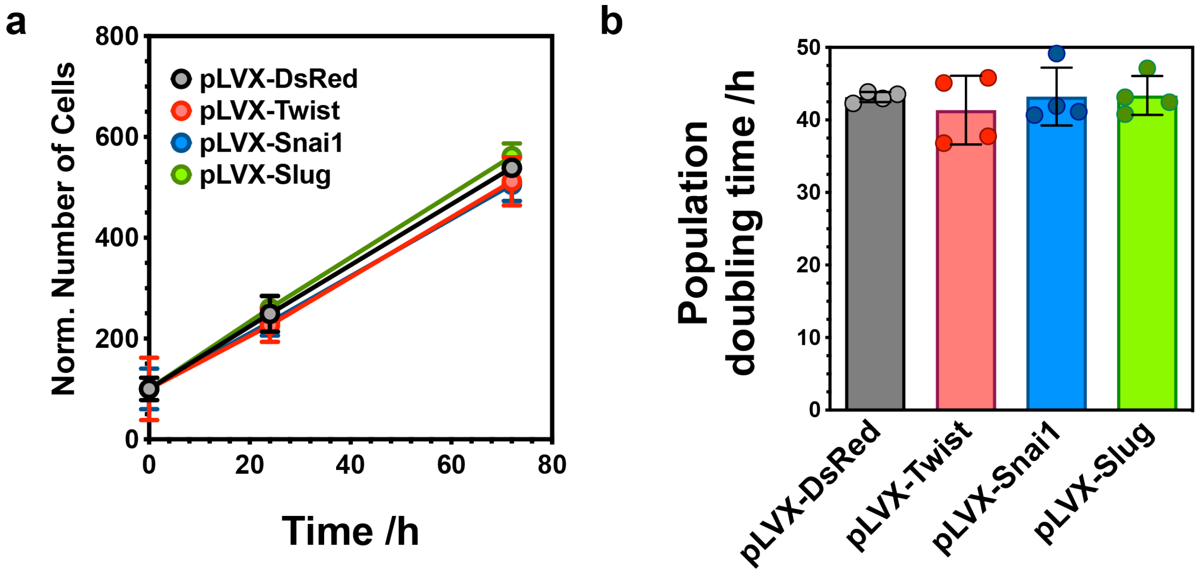


**(a)** Cell proliferation of HUVEC stably overexpressing Twist1, Slug, or Snai1 over 72h in comparison to HUVEC transfected with the control lentiviral vector (pLVX-DsRed).

**(b)** Cell doubling time of HUVEC stably overexpressing Twist1, Slug, or Snai1 in comparison to HUVEC transfected with the control lentiviral vector (pLVX-DsRed).

**Supplemental Figure 3**

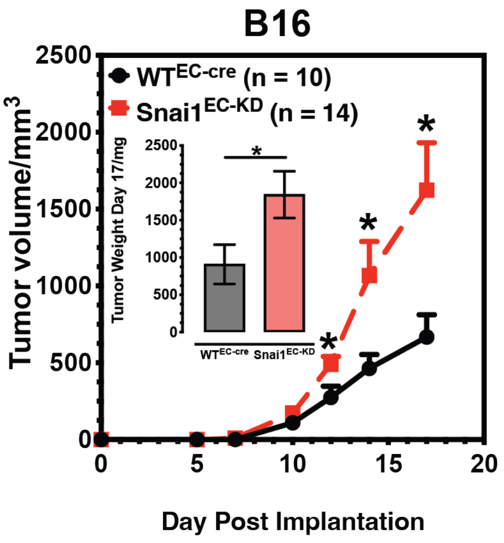


Growth rate of B16 melanoma is increased by implantation into Snai1$^{EC-KD}$ mice.

## Supplemental Figure 4

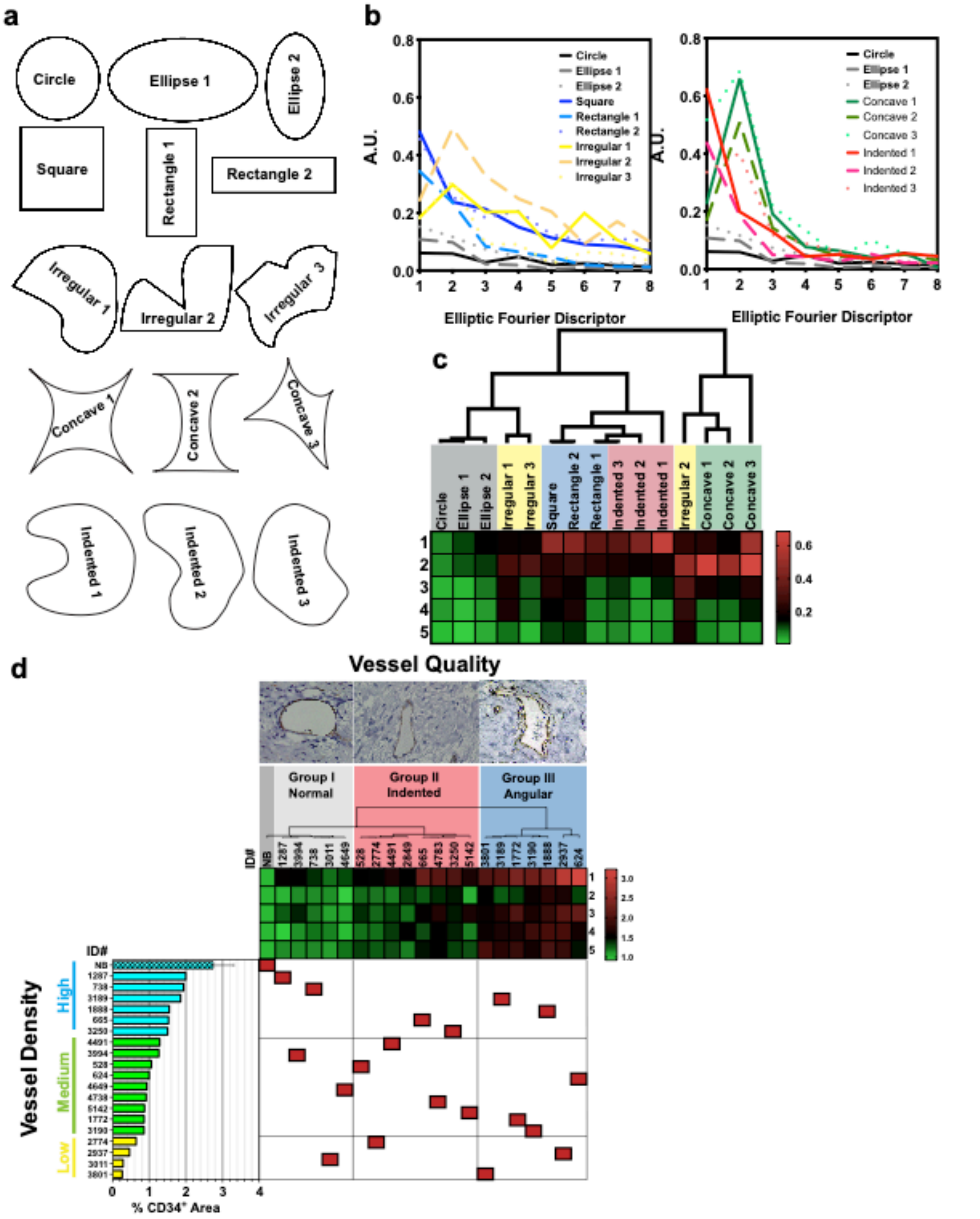


**(a)** Test shapes to establish Elliptic Fourier Analysis (EFA) as a method to rank vascular irregularities in archival tissue sections.

**(b)** Results from EFA for the test shapes shown in (a). Displayed are the first eight Fourier descriptors (FD). For clarity the data is divided in two graphs.

**(c)** Cluster analysis of the five first FD for the test shapes shown in (a).

**(d)** Results from elliptic Fourier analysis (EFA) of blood vessels in human breast cancer sections. Vessels in CD34 stained sections were evaluated by EFA, and the results were compared to normal breast tissue (N.B.) and clustered according to the first five elliptic Fourier descriptors. The three emerging phenotype clusters were named *normal*, *indented*, and *angular* according to their co-clustering with vessels in normal breast tissue or their predominant appearance. A correlation of vessel phenotype with vessel density (by $CD34^{+}$-area) is not apparent.

**Supplemental Figure 5**

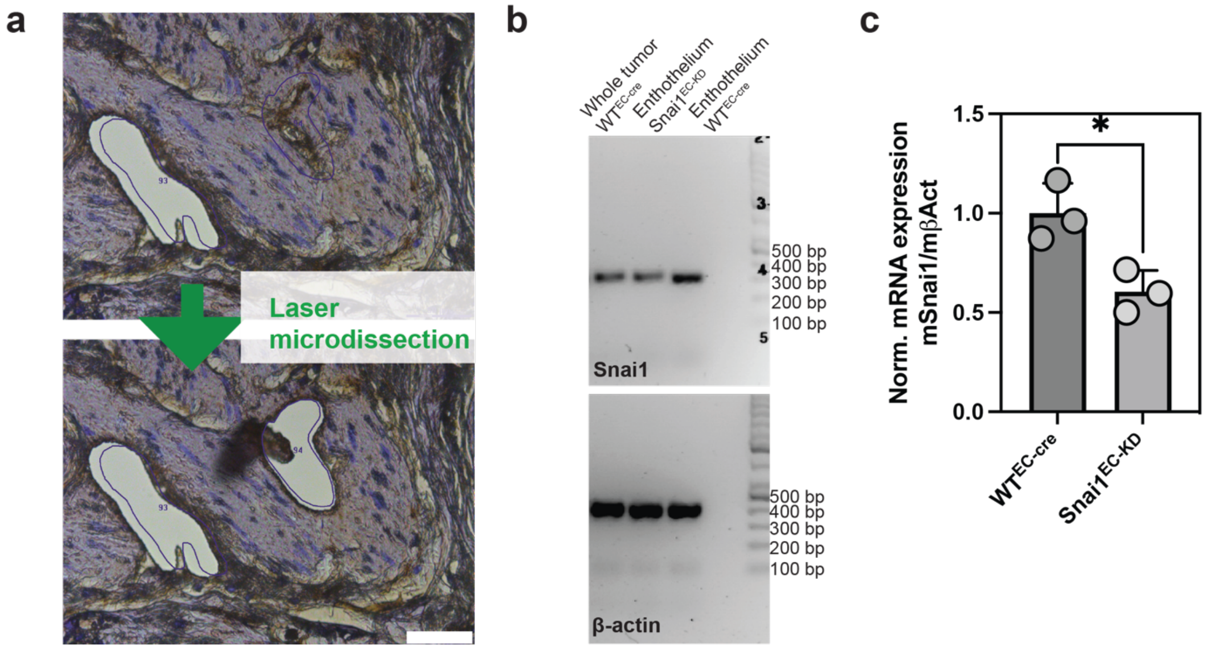


Validation of reduced Snai1-mRNA expression in the tumor endothelium of $Snai1^{EC-KD}$ mice

**(a)** Images of a tumor section stained for CD34 with blood vessels marked for laser dissection. In the upper panel, one vessel is already dissected, and a second is marked. In the lower panel, the second vessel structure is also cut out. Scale bar = 50 µm.

**(b)** RT-PCR results from laser microdissected LLC tumor samples after RNA extraction and amplification. In addition to $CD34^+$-endothelium, small samples of the general tumor tissue were also microdissected as a control (first lane).

**(c)** Quantification of Snai1-mRNA content in laser microdissected $CD34^+$-endothelium from tumor grown in $WT^{EC-cre}$ or $^{Snai1EC-KD}$ mice by RT-qPCR.

**Supplemental Figure 6**

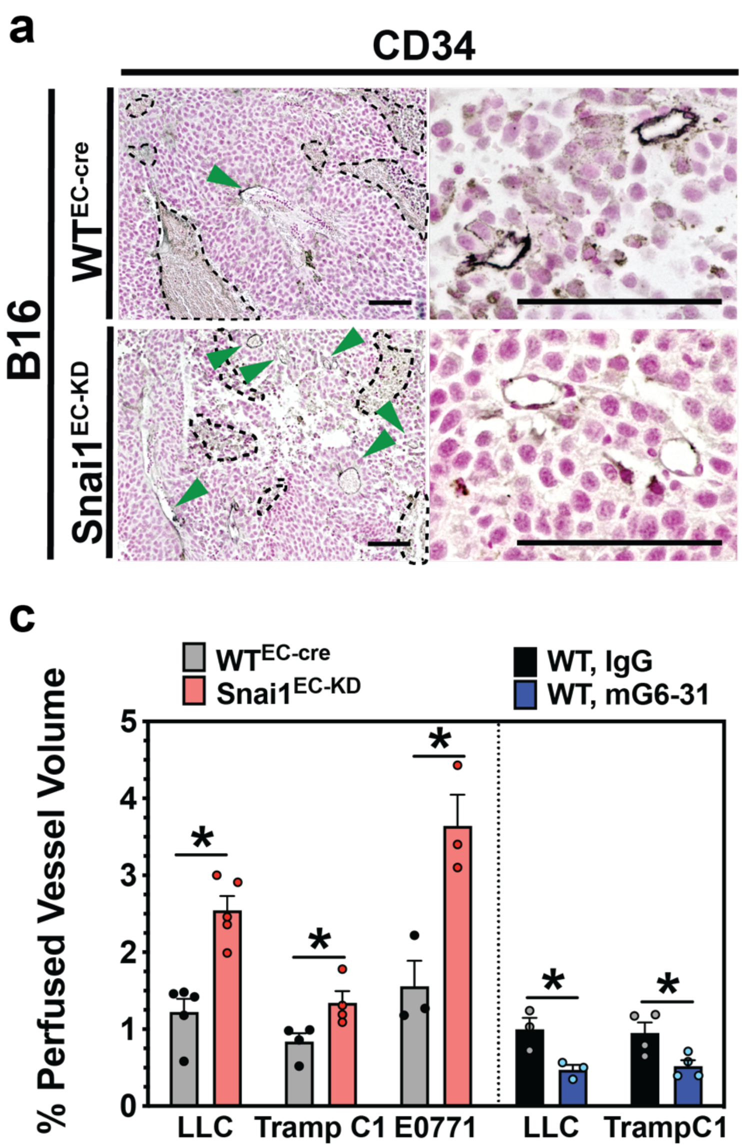


**(a)** Analysis of blood vessels in sections of implanted B16 murine melanomas grown in WT$^{EC\text{-}cre}$ and Snai1$^{EC\text{-}KD}$ mice by IHC (vascular marker CD31).

**(b)** Microvessel density (MVD) evaluated in CD31 IHC stained B16 murine melanoma sections. MVD is significantly increased in Snai1$^{EC\text{-}KD}$ mice.

**(c)** Evaluation of the relative volume of perfused vessels in various murine tumors. Endothelial-specific knock-down of Snai1 increased vessel volume, while VEGF-A sequestration reduced it. (n = 4-5)

**Supplemental Figure 7**

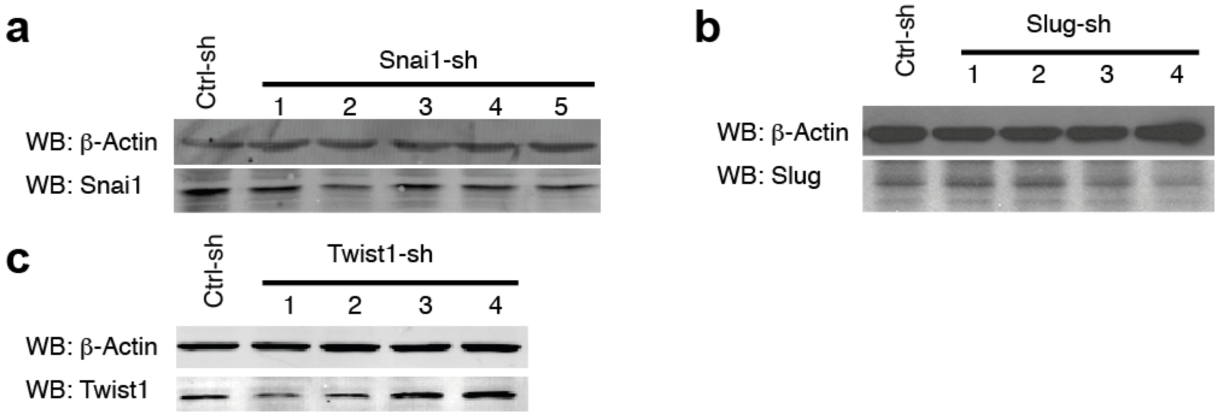


**(a)** Western blot analysis of Snai1-expression in HUVEC stably transfected with 5 different shRNA-transferring lentiviral particles. HUVEC transfected with Snai1-shRNA-2 and Snai1-shRNA-3 were used for functional assays as they produced the strongest reduction of Snai1 protein levels.

**(b)** Western blot analysis of Slug-expression in HUVEC stably transfected with 4 different shRNA-transferring lentiviral particles. HUVEC transfected with Slug-shRNA-3 and Slug-shRNA-4 were used for functional assays as they produced the strongest reduction of Slug protein levels.

**(c)** Western blot analysis of Twist1-expression in HUVEC stably transfected with 4 different shRNA-transferring lentiviral particles. HUVEC transfected with Twist1-shRNA-1 and Twist1-shRNA-2 were used for functional assays as they produced the strongest reduction of Twist1 protein levels.
